# Supplementary material for: Crystal structures of the human elongation factor eEFSec suggest a non-canonical mechanism for selenocysteine incorporation
Source: Nat Commun. 2016 Oct 6;7:12941. doi: 10.1038/ncomms12941 (PMC5059743; doi:10.1038/ncomms12941)
Supplement: Supplementary Information — Supplementary Figures 1-7 and Supplementary References. [file ncomms12941-s1.pdf]

Supplementary Figure 1

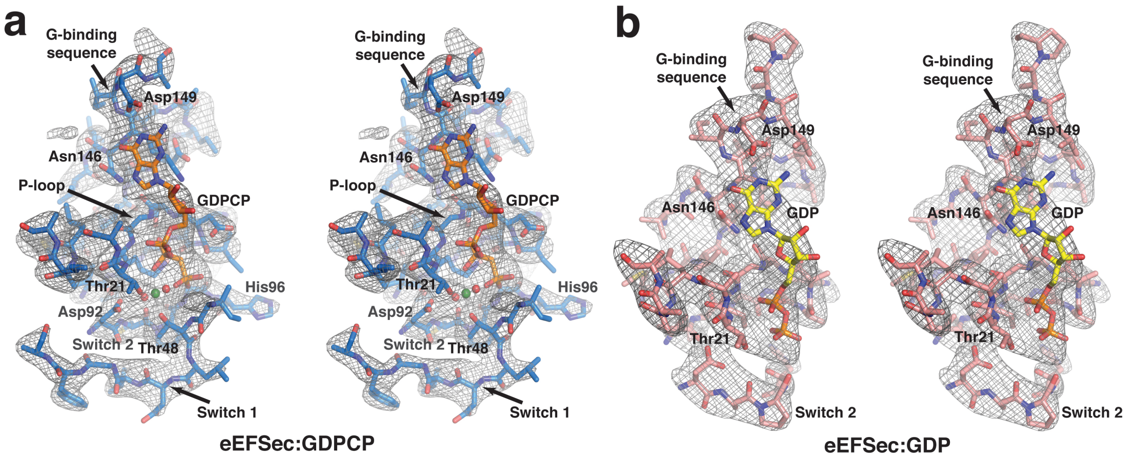

The stereo-view of the final models of eEFSec and the corresponding electron density maps. **(a)** The GTPase site of the GTP-bound eEFSec. GDPCP is shown as orange sticks, while the eEFSec residues are shown as blue sticks. The final 2mFo-DFc electron density difference map (grey mesh) is calculated to 2.7 Å and contoured at 2σ. Water molecules and Mg^{2+} are red and green spheres, respectively. **(b)** The GTPase site of eEFSec (pink sticks) complexed with GDP (yellow sticks). The view is oriented the same as in (a). The final 2mFo-DFc electron density map (grey mesh) is calculated to 3.4 Å and contoured at 2σ. The main elements of the GTPase site are labeled as in the text.

Supplementary Figure 2

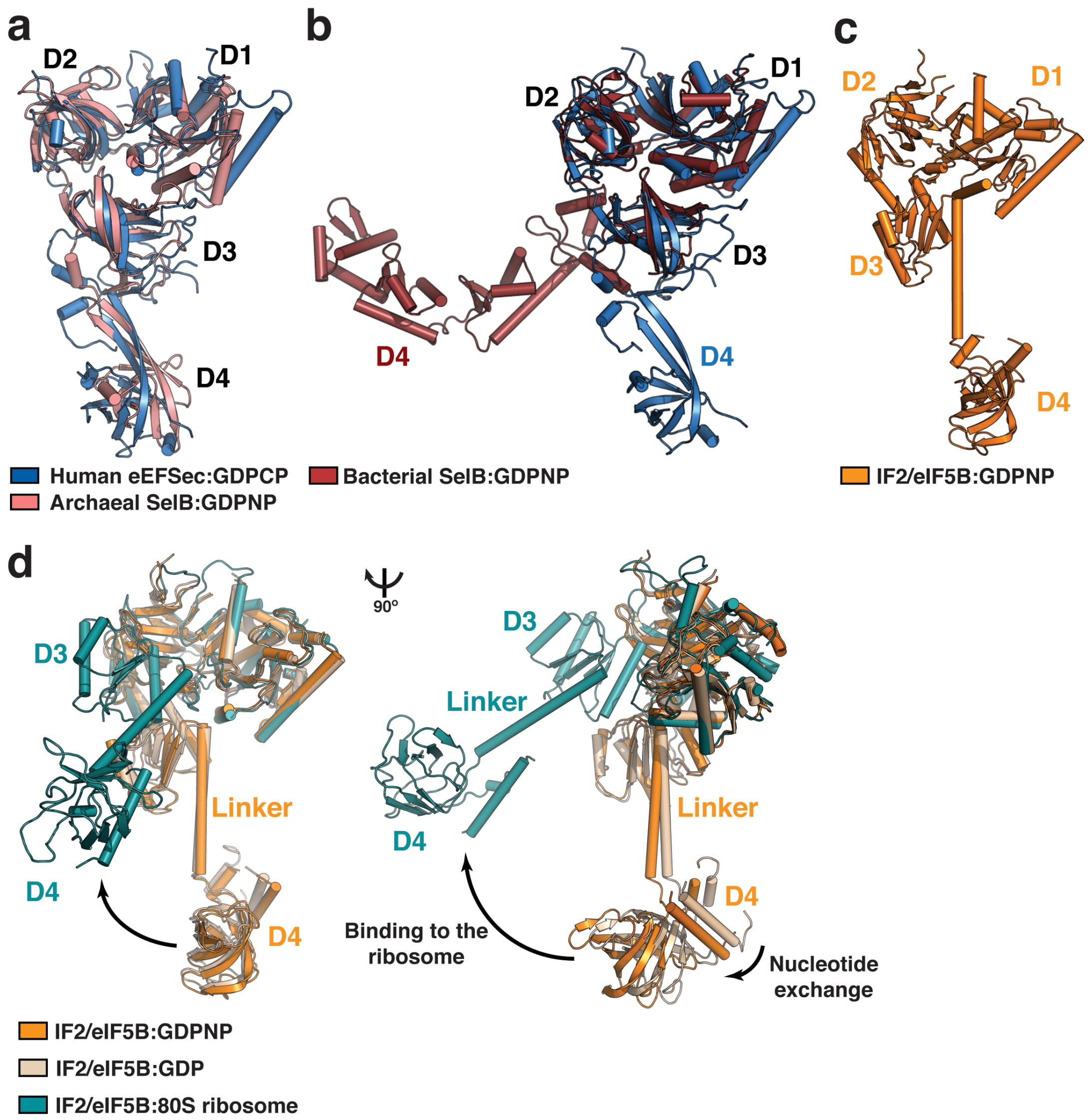

Human eEFSec is similar to the archaeal SelB, and resembles the bacterial SelB and IF2/eIF5B. The global superimpositioning of human eEFSec:GDPCP (blue) onto archaeal SelB:GDPNP (**a**; pink) and bacterial SelB:GDPNP (**b**; dark red), reveals that molecules adopt similar structures with the most marked differences present in the appended domain D4. **(c)** The overall domain arrangement and shape of eEFSec and the archaeal IF2/eIF5B (orange) are similar. **(d)** The overlay of the main functional states of IF2/eIF5B reveals dynamics of D4 and D3. D4 undergoes a slight translation upon nucleotide exchange, followed by a ~50° rotation of D3, linker and D4 after binding to the 80S ribosome and the A-site tRNA. The GDP-bound state is beige, the GTP-bound state is orange, and the ribosome-bound conformation of IF2/eIF5B is teal [1,2]. Two views are related by ~90° clockwise rotation around the vertical axis. The view on the left is similar to that in (c).

Supplementary Figure 3

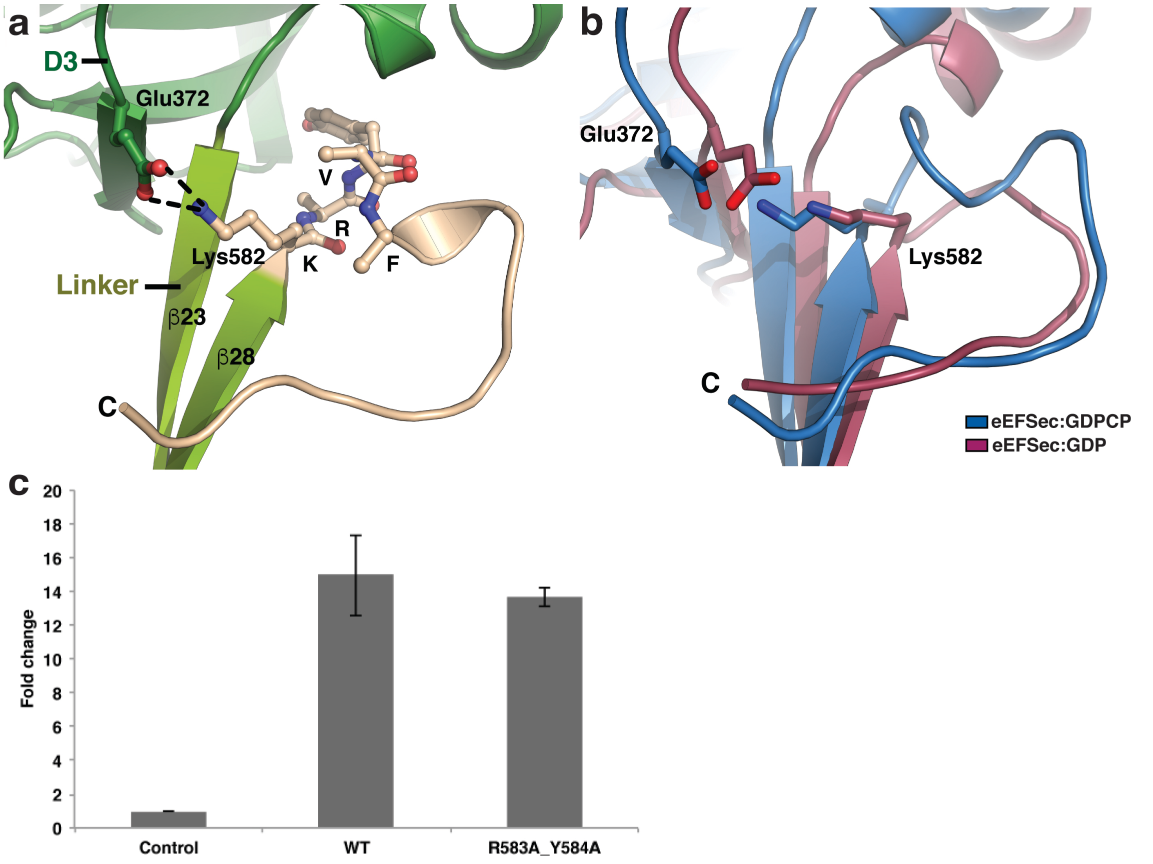

The structure of the "hinge" region in human eEFSec. **(a)** The "hinge" is at the interface between D3 (dark green), the linker region (light green), and the C-terminal segment (beige). The side chain of the conserved Glu372 (dark green sticks) forms H-bonds with the side chain of Lys582 (beige sticks) from the "KRVYF" motif (beige sticks), which connects strand β28 of the linker with the C-terminal segment. The salt bridge between Glu372 and Lys582 is conserved in the archaeal SelB, but absent from the bacterial ortholog. This interaction may be significant for stabilizing the interdomain orientation in eEFSec. The side chains of Arg583, Val585, and Phe586 in "KRYVF" are disordered in our crystals. **(b)** The salt bridge between Glu372 and Lys582 is preserved in the GDP- (red) and GDPCP (blue) structures of eEFSec. H-bonds and other side chains of the "hinge" region are not shown for clarity. The view is oriented similar to that in (a) and the coloring scheme is the same as in Fig. 2. **(c)** The double ^{583}RY^{584} -> AA mutation within the 'hinge' region does not impair the Sec incorporation activity of eEFSec *in vitro*. Error bars represent standard deviation calculated from three replicates.

Supplementary Figure 4

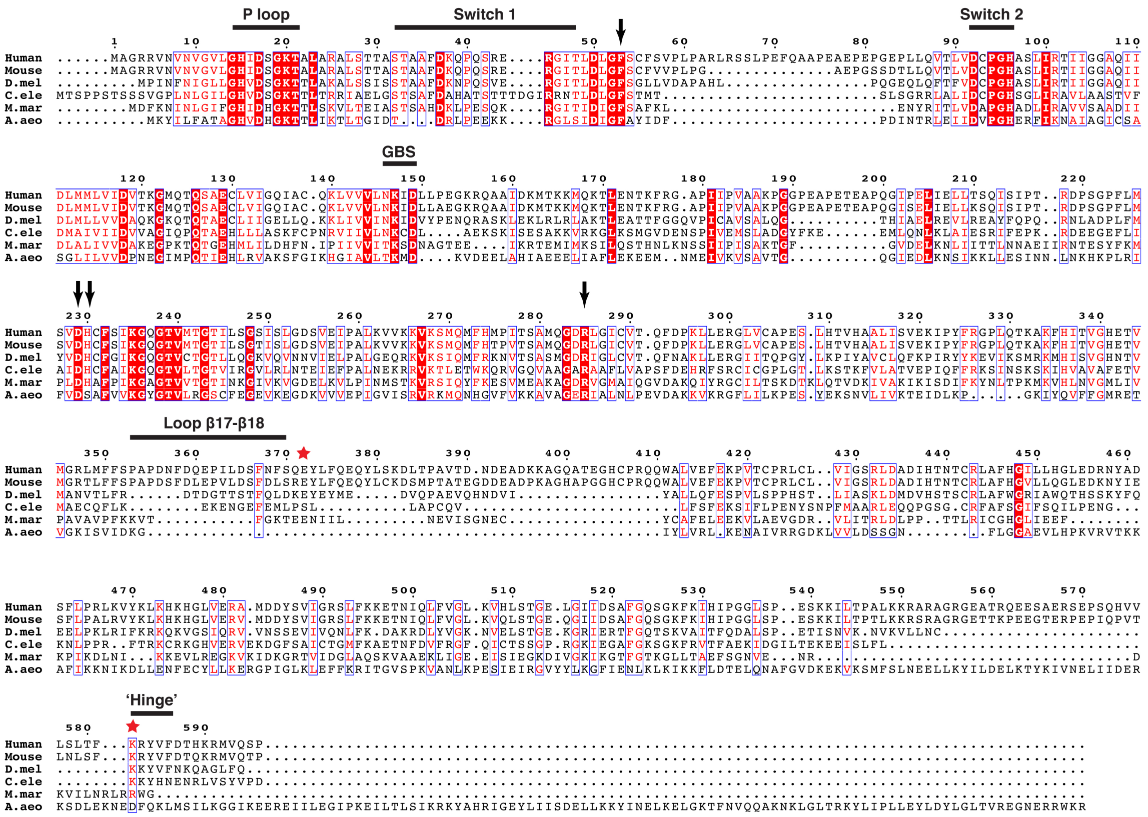

Structure-based sequence alignment of eEFSec and SelB orthologs. The components of the GTPase site (i.e. P loop, Switch 1, Switch 2, and guanine-binding sequence) and the "KRYVF" sequence of the 'hinge' region are highlighted with black bars. The guanine-binding sequence is labeled as "GBS". Arrows point to residues of the selenocysteine-binding pocket, and red stars highlight the ^{372}Glu-Lys^{582} salt bridge in the 'hinge'. Sequences were aligned using MultAlin [3] and the figure was prepared using ESPript 3.0 [4]. D.mel: *Drosophila melanogaster*, C.ele: *Caenorhabditis elegans*, M.mar: *Methanococcus maripaludis*, A.aeo: Aquifex aeolicus.

Supplementary Figure 5

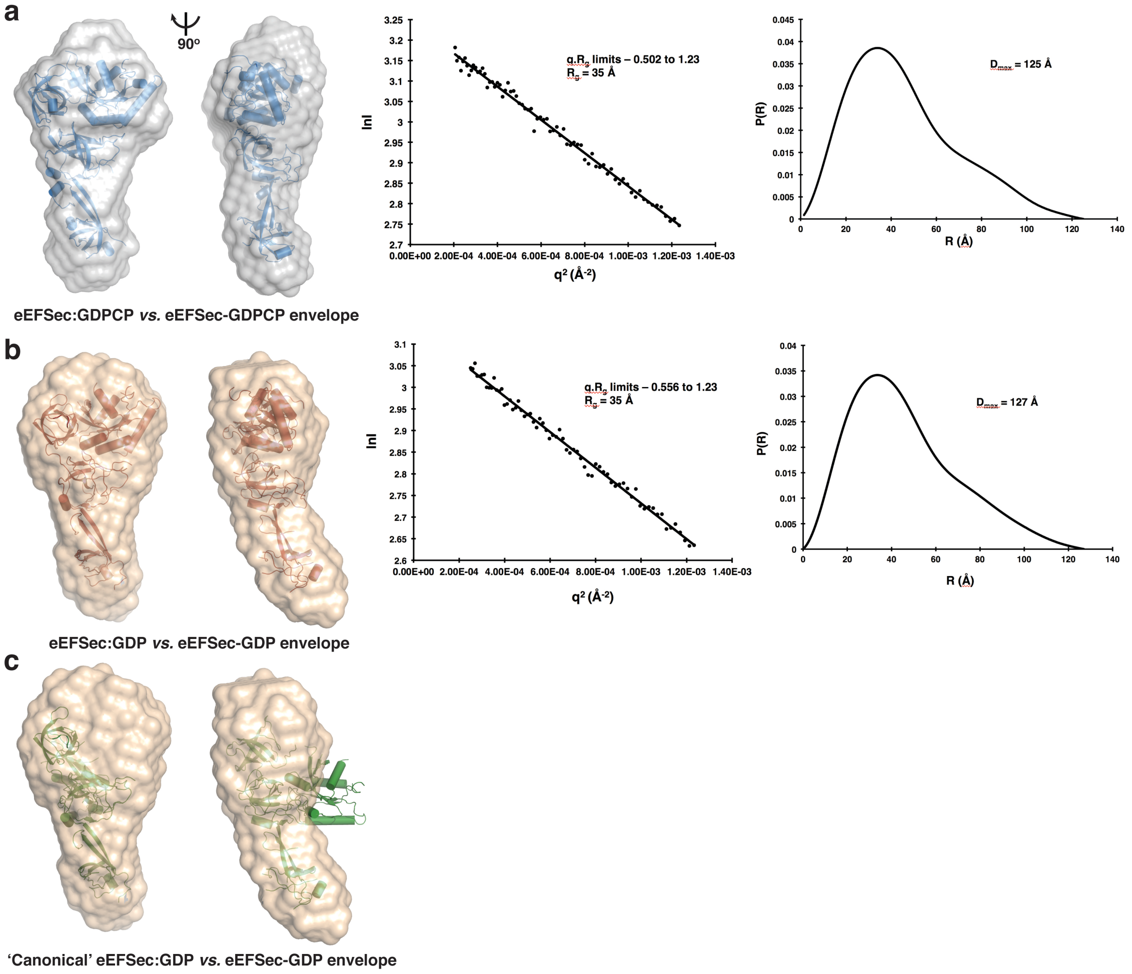

The conformational change between the GDP and GTP states observed in eEFSec in crystals also occurs in solution. **(a)** The eEFSec:GDPCP crystal structure (blue cartoon) agrees well with its SAXS envelope (gray surface). The corresponding Guinier (middle panel) and P(R) plots (right panel) show that eEFSec is a multidomain protein with Rg of 35 Å. **(b)** The structure of the GDP-bound eEFSec (red cartoon) superimposes onto the eEFSec-GDP envelope (beige surface). The Guinier (middle) and P(R) plots (right panel) reveal that Rg of eEFSec does not alter upon nucleotide exchange. **(c)** The 'canonical' model of the GDP-bound eEFSec (green cartoon; see the main text for detail) does not fit into the envelope of eEFSec:GDP (beige surface); the canonical domain arrangement places D1 outside of the molecular envelope. Two views related by ~90° clockwise rotation around the vertical axis are shown. The EF-Tu-like domain is kept in a similar orientation in all panels.

Supplementary Figure 6

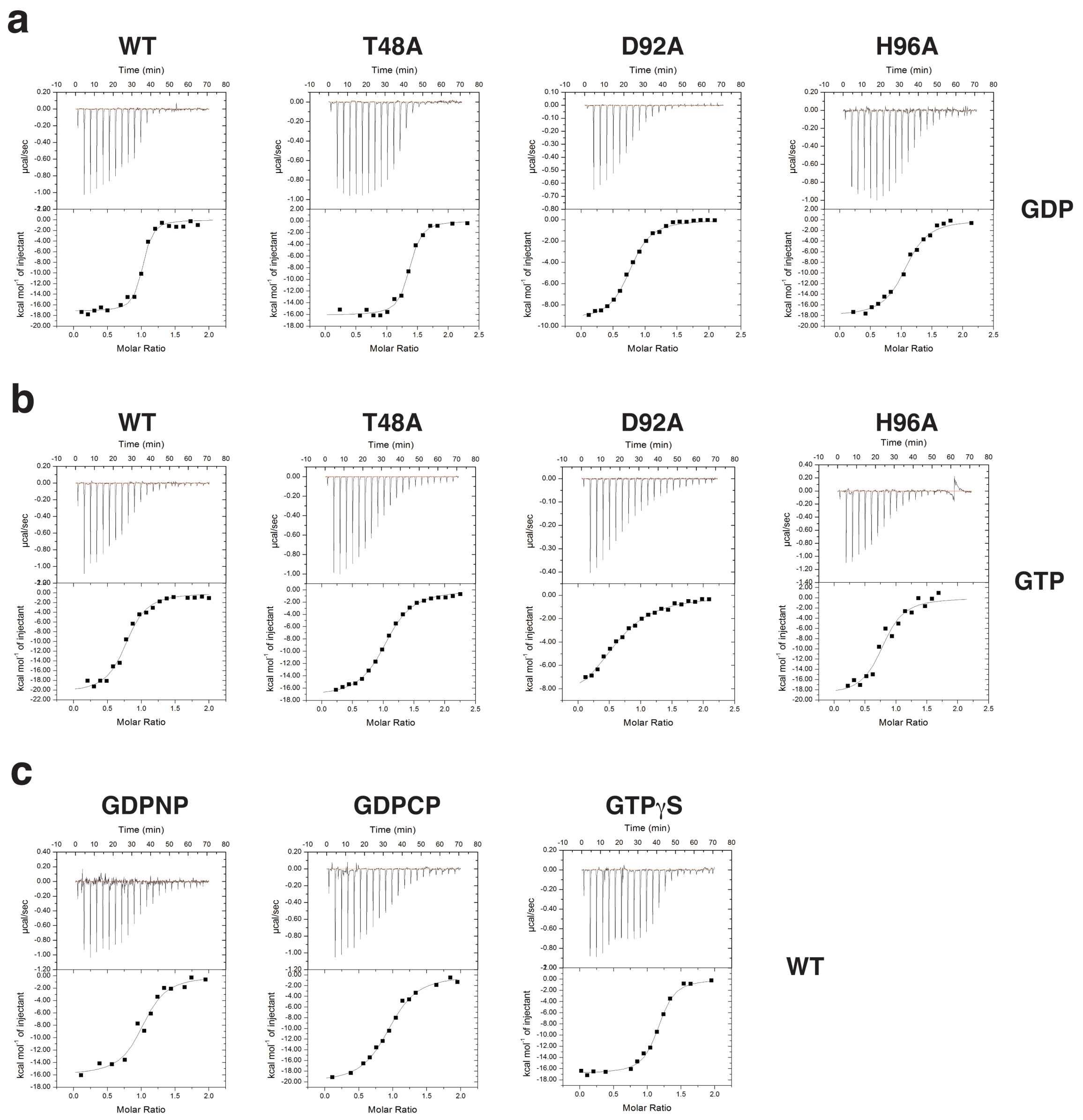

The binding of guanine nucleotides and analogs to the WT and GTPase site variants of human eEFSec. The titration curves (upper panels) and binding isotherms (lower panels) derived from interactions of eEFSec with GDP **(a)**, GTP **(b)**, and non-hydrolyzable GTP analogs **(c)** are shown. The thermodynamic parameters calculated from the ITC data are in **Table 2**.

Supplementary Figure 7

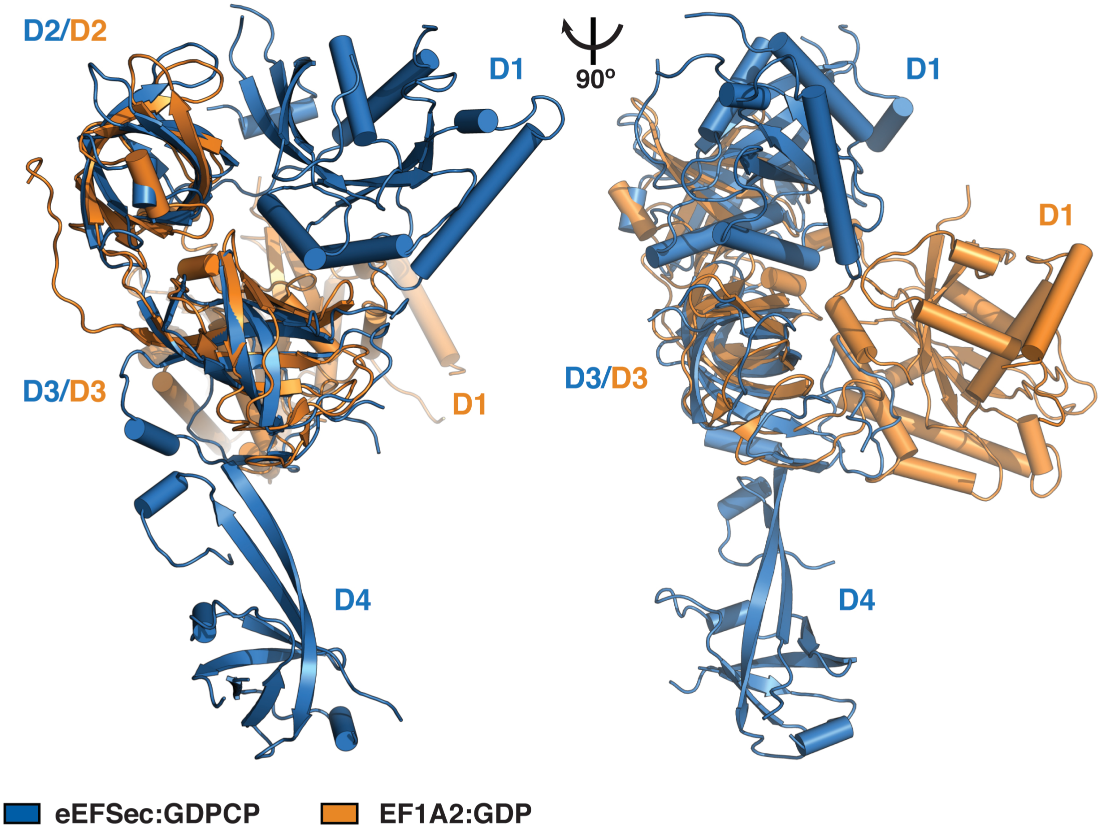

The superimpositioning of human eEFSec:GDPCP (blue) onto rabbit EF1A2:GDP (orange) reveals different arrangement of D1-3. This further confirms that GDPCP and GDPNP trap eEFSec in the GTP-bound state. Two views are rotated ~90° clockwise around the vertical axis.

Supplementary References:

1. Roll-Mecak, A., Cao, C., Dever, T.E. & Burley, S.K. X-Ray structures of the universal translation initiation factor IF2/eIF5B: conformational changes on GDP and GTP binding. *Cell* **103**, 781-92 (2000).
2. Fernandez, I.S. et al. Molecular architecture of a eukaryotic translational initiation complex. *Science* **342**, 1240585 (2013).
3. Corpet, F. Multiple sequence alignment with hierarchical clustering. *Nucleic Acids Res* **16**, 10881-10890 (1988).
4. Robert, X. & Gouet, P. Deciphering key features in protein structures with the new ENDscript server. *Nucleic Acids Res* **42**, W320-W324 (2014).
